# Turning over New Leaves

**Published:** 1889-03-16

**Authors:** 


### TURNING OVER NEW LEAVES.

(Books for Review should be sent to The Editor. The Lodge, Porchester Square, W.)

### Something Wonderful.*

THIS book is a curiosity in its way. No one can read it without feeling an intense surprise that it ever found a publisher. There are a number of stories in the volume, but practically they are all the same: There was once a deserving but impecunious young man. Sometimes he is a curate, sometimes a clerk, but as he always tells the tale in the first person, you havn't even a frequently-recurring name to guide you, and get hopelessly confused as to which of the narratives you were reading if you happen to lay down the book for a moment. This deserving young man meets a young and beautiful heiress with charming parents, who are only too delighted to wed their daughter to the deserving and impecunious one. The situation is a pleasing one, especially to love lorn bachelors who have not yet met with such amiable heiresses, but after reading it over half-a-dozen times with scarcely a jot of difference, it will become monotonous even to them. Even a reader who is not passionately fond of sensation might incline to a moderate indulgence in Rider Haggard or Gaborian, after he had got to the end of that eminently-soothing narrative which is erroneously entitled "The Strangest Journey of My Life," and which makes one realise enviously how very placid Mr. Pigott's life must have been. The style is easy, not to say slipshod. Why any man says "did not have," when it is within his rights as an Englishman to say "had not," must always remain a puzzle to the critic.

### New Music.

A SONG lies on our table, called "Re-united," by "Torriano" (Hart and Co., 22, Paternoster Row, E.C.). The words are by "W. H. H. M.," and are pretty and suitable, though the subject is not very original. The music is, on the whole, pleasing, each verse ending with a refrain in 3-4 time, the remainder being in common time. Should the writer be tempted to publish further essays of the kind, it is to be hoped that more care will be taken to avoid technical and grammatical errors. The composer shows a fine contempt for the conservative fads of harmony, which forbid the use of consecutive fifths, parallel sequences, and others which grate harshly on the ear. All these militate heavily against a song, but those whose musical education is simple enough to enable them to overlook such little matters will find "Re-united" pretty enough, and really not so far behind some of the ballads which flood our drawing-rooms and lighter concert-halls.

*"The Strangest Journey of My Life." By F. C. Pigott: London, Ward and Downey.

